# Significant Improvement in Diagnosis of Hepatitis C Virus Infection by a One-Step Strategy in a Central Laboratory: an Optimal Tool for Hepatitis C Elimination?

**DOI:** 10.1128/JCM.01815-19

**Published:** 2019-12-23

**Authors:** Rosa López-Martínez, Andrea Arias-García, Francisco Rodríguez-Algarra, Laura Castellote-Bellés, Ariadna Rando-Segura, Guillermo Tarraso, Elena Vargas-Accarino, Isabel Montserrat-Lloan, Albert Blanco-Grau, Andrea Caballero-Garralda, Roser Ferrer-Costa, Tomas Pumarola-Sunye, Maria Buti-Ferret, Rafael Esteban-Mur, Josep Quer, Ernesto Casis-Saez, Francisco Rodríguez-Frías

**Affiliations:** a Department of Clinical Biochemistry (Clinical Laboratories), University Hospital Vall d’Hebron, Barcelona, Spain; b Clinical Biochemistry Research Group, Vall d’Hebron Institute of Research (VHIR), Barcelona, Spain; c Blizard Institute, Barts and the London School of Medicine and Dentistry, Queen Mary University of London, London, United Kingdom; d PROSICS Barcelona, Microbiology, University Hospital Vall d’Hebron, Barcelona, Spain; e Department of Haematology and Haemotherapy, University Hospital Vall d’Hebron, Barcelona, Spain; f Department of Internal Medicine and Hepatology, University Hospital Vall d’Hebron, Barcelona, Spain; g Liver Unit-Internal Medicine, Centro de Investigación Biomédica en Red de enfermedades hepáticas y digestivas (CIBEREHD), Carlos III Institute, Madrid, Spain; h Department of Microbiology (Clinical Laboratories), University Hospital Vall d’Hebron, Barcelona, Spain; i Bioscience and Medicine Schools, Autonomous University of Barcelona (UAB), Barcelona, Spain; Cepheid

**Keywords:** hepatitis C virus, diagnosis, reflex testing, viral load, one-step diagnosis

## Abstract

The remarkable effectivity of current antiviral therapies has led to consider the elimination of hepatitis C virus (HCV) infection. However, HCV infection is highly underdiagnosed; therefore, a global strategy for eliminating it requires improving the effectiveness of HCV diagnosis to identify hidden cases. In this study, we assessed the effectiveness of a protocol for HCV diagnosis based on viral load reflex testing of anti-HCV antibody-positive patients (known as one-step diagnosis) by analyzing all diagnostic tests performed by a central laboratory covering an area of 1.5 million inhabitants in Barcelona, Spain, before (83,786 cases) and after (45,935 cases) the implementation of the reflex testing protocol. After its implementation, the percentage of anti-HCV-positive patients with omitted HCV RNA determination remarkably decreased in most settings, particularly in drug treatment centers and primary care settings, where omitted HCV RNA analyses had absolute reductions of 76.4 and 20.2%, respectively. In these two settings, the percentage of HCV RNA-positive patients identified as a result of reflex testing accounted for 55 and 61% of all anti-HCV-positive patients. HCV RNA results were provided in a mean of 2 days. The presence of HCV RNA and age of ≥65 years were significantly associated with advanced fibrosis, assessed using the serological FIB-4 index (odds ratio [OR], 5.92; 95% confidence interval [CI], 3.4 to 10.4). The implementation of viral load reflex testing in a central laboratory is feasible and significantly increases the diagnostic effectiveness of HCV infections, while allowing the identification of underdiagnosed cases.

## Abstract (precis)

The remarkable effectivity of current antiviral therapies has led to consider the elimination of hepatitis C virus (HCV) infection. However, HCV infection is highly underdiagnosed; therefore, a global strategy for eliminating it requires improving the effectiveness of HCV diagnosis to identify hidden cases.

## INTRODUCTION

Hepatitis C virus (HCV) infection is a major health problem worldwide. The overall prevalence of chronic HCV infection is estimated to amount to 71 million people (1). Still, HCV infection continues to be considered an underdiagnosed disease, and it is estimated that about half of the people infected might not be aware of it (2, 3). Based on prevalence data from Spain, there might be over 200,000 cases of undiagnosed HCV infections (4).

Last-generation direct-acting antivirals (DAAs) have reached cure rates of over 95% and provide more benefits when the disease is treated in its initial stages (5, 6). These outstanding improvements in therapeutic tools have driven the proposal of strategies to eradicate the HCV infection. Based on the initiative by the World Health Organization, it is critical to identify infected individuals both to start therapy promptly and to avoid spreading and the occurrence of new cases (7). However, in daily clinical practice, the diagnostic process of HCV infections and patients’ hospital referrals usually require multiple visits, which delays the diagnosis and increases the risk to lose the patient to follow-up (8).

In our area (Catalonia, northeast Spain), *in vitro* diagnoses provided by the public health care system, covering over 80% of a population of 7.5 million people, are centralized in six laboratories (9). Ours, the largest in Spain, centralizes HCV-related analytical tests from the entire primary care setting of a city with 1.5 million people (Barcelona), plus several public health facilities, thus covering most entry points for new HCV infections. This remarkable coverage offers an opportunity to increase the rate of hepatitis C diagnosis by implementing new strategies driven by the laboratory.

**FIG 1:**
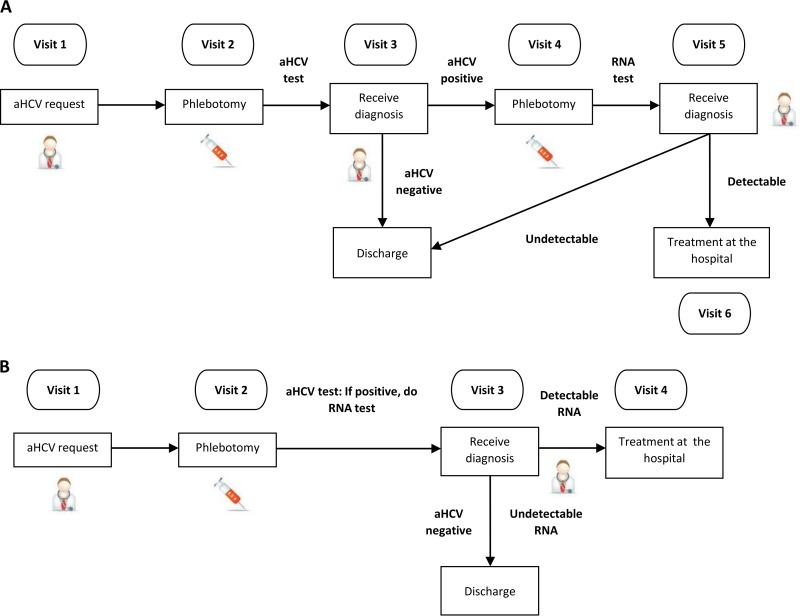
HCV infection diagnosis flowchart in an ambulatory setting before (A) and after (B) the implementation of the reflex-testing-based protocol or one-step protocol. aHCV, anti-hepatitis C virus antibody; VL, viral load.

Traditionally, HCV screening in outpatients starts with the request of antibodies against HCV (anti-HCV) by physicians; in case of positive anti-HCV results, the physician should request a new blood draw to test viral load (Fig. 1A). The medical visit and blood extraction cannot be done on the same day because they are performed in different centers: blood extractions are performed by specialized phlebotomists located at specific sites, which centralize different medical consultations and require a prior appointment. In addition to be very upsetting for the patient, this complex process is highly prone to end up with a loss to follow-up of infected patients. In fact, a remarkable proportion of anti-HCV-positive cases in our laboratory remain untested for viral load. To reduce lost-to-follow-up rates, in 2015 our laboratory introduced a strategy to promote viral load requests in new cases of patients who tested positive for anti-HCV antibodies through comments in the analytical report. However, this action did not meet our expectations. Therefore, following the recommendations of the European Association for the Study of the Liver (EASL) (10), we implemented a one-step diagnostic protocol, known as reflex testing, in which the sample collected for blood count analysis is used to determine HCV RNA in anti-HCV-positive patients (Fig. 1B), eliminating the need for the physician to request the test and notably reducing the number of visits. In recent years, similar strategies aimed at reducing the number of visits needed for diagnosis have been described (8, 11); however, there is very little information on the impact of its implementation on the diagnosis of new cases. Strategies aimed at optimizing screening programs seem to be essential to eradicate HCV infections, as has been set as an objective by the World Health Organization (2).

This study presents the results of both screening protocols and a comparison of their effectiveness at detecting new cases of HCV infection. Furthermore, considering that the sequential use of a blood sample for blood count and HCV load analyses is a key element of the one-step protocol, the integrity and suitability of viral RNA were also assessed. Finally, and since the data obtained allowed determination of the degree of fibrosis using the FIB-4 score, its relationship with viral load was explored.

## MATERIALS AND METHODS

### Study design and diagnostic protocols.

**FIG 2:**
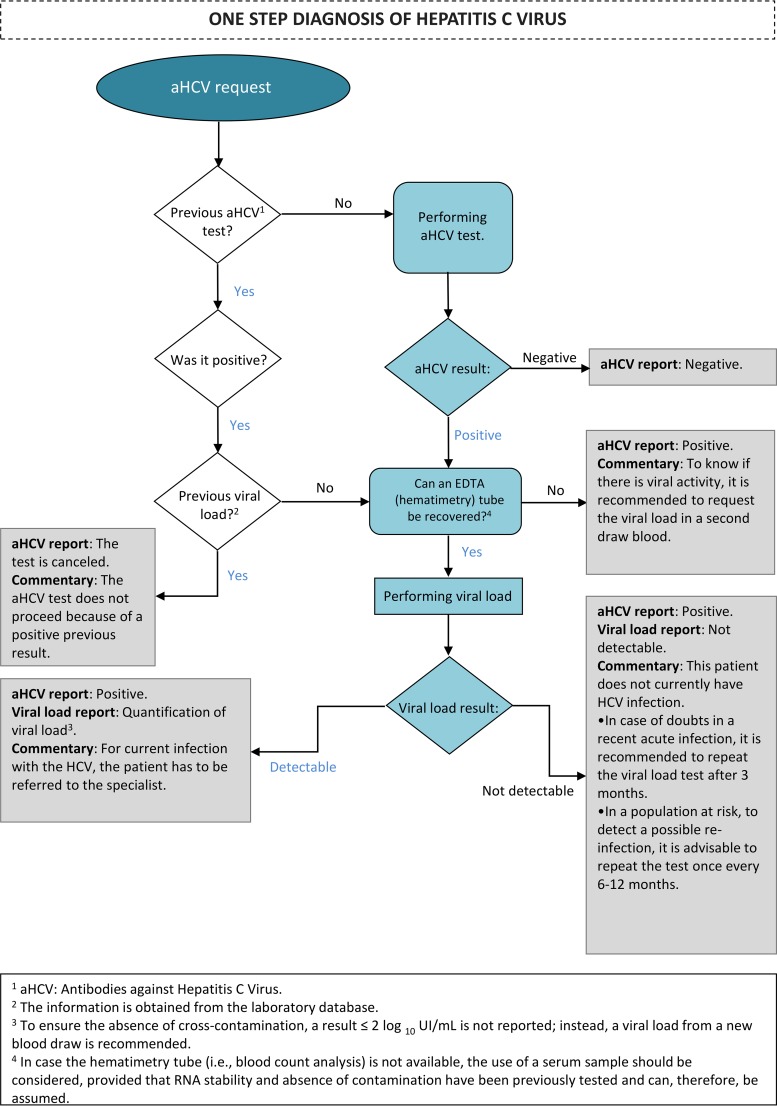
Detailed flowchart of the reflex-testing-based (one-step) protocol for HCV infection diagnosis.

This study was an analysis of all HCV diagnostic tests recorded in the laboratory information system (LIS) of Vall d’Hebron Barcelona Hospital (Spain) between January 2015 and December 2018. The laboratory centralizes the analytical tests from a 1,200-bed hospital, as well as all samples coming from primary care centers and other public facilities of Barcelona (1.5 million inhabitants). The study period encompasses two different diagnostic protocols: before March 2018, we actively recommended to perform HCV RNA determination in anti-HCV-positive outpatients upon prior request by the health care provider (six-step protocol) (Fig. 1A); whereas from March 2018, the laboratory used the whole-blood EDTA sample collected for blood count analysis (hematimetry [hemocytometry]) to determine the HCV RNA in all anti-HCV-positive patients (reflex testing or one-step protocol) (Fig. 1B). Results of the HCV RNA test were provided within 24 h after anti-HCV testing, on Monday to Thursday, and within 3 days in case the anti-HCV test was performed on a Friday. Serological testing (anti-HCV determination) was performed on the Cobas 8000 platform (Roche Diagnostics, Germany) with single-use (i.e., disposable) tips. In both protocols, the determination of HCV RNA was performed with a Cobas 6800 analyzer (Roche Diagnostics, Germany); the blood count analysis prior to determination of HCV RNA was performed by flow cytometry with a Sysmex XN-20 analyzer (Sysmex Corporation, Japan). Therefore, the sample used for HCV RNA had been previously used for hematimetry studies. However, the Sysmex XN-20 analyzer can perform hematimetric measures without decapping blood tubes (the rubber cap is punched by a needle). The potential use of serum samples previously collected for serology was initially ruled out due to the higher risk of carryover. First, since the analyzer centralizes serological determinations, the system tends to concentrate samples with suspected hepatitis C (i.e., is potentially anti-HCV positive). Second, although the Cobas 8000 analyzer uses disposable tips, samples reach the analytical system uncapped and they are, therefore, exposed to environmental aerosols and other occasional hand manipulations, which may increase the risk of contamination. In contrast, all hematimetry determinations are randomly processed (more than 5,000 a day), meaning that potential anti-HCV-positive cases (about 5 to 10 a day) would be randomly distributed among these 5,000 samples, statistically reducing the probability of cross-contamination associated with that of two anti-HCV-positive cases: one positive HCV RNA located near, and prior to, one negative HCV RNA. In spite of this, a cross-contamination study was performed to ensure the quality and adequacy of this type of sample. Said HCV RNA analysis is only carried out in those cases lacking any data prior to the determination of the viral load (see the algorithm in Fig. 2).

### Assessment of diagnostic protocols.

The result of adding a recommendation to perform a HCV RNA analysis in anti-HCV-positive outpatients was assessed using the data from the medical history of non-hospital patients.

In order to assess and compare the effectiveness of both protocols, all cases from the following periods were considered: January 2017 to February 2018 for the six-step protocol (82,786 cases), and March 2018 to December 2018 after implementing the reflex-testing-based protocol for HCV RNA (45,935 cases). The causes for omitting requests were assessed based on the information contained in the patient’s medical history. Cases were classified based on the source of the request: primary care (i.e., general population attended in community health centers), drug treatment centers (i.e., outpatient centers for drug dependence care and follow-up [“CAS” according to its initials in Spanish]), hospital care (hospitalized patients, hospital consultations, and an external site specializing in imported and sexually transmitted diseases), itinerant blood samples (from patients who, due to different reasons, are sequentially or even simultaneously treated by different units and thus shared by the different groups mentioned above), and other sources (prisons and children’s care homes).

### Study of sample suitability and integrity.

To assess the absence of sample contamination, a carryover study of HCV RNA was conducted with samples already used for blood count performed by flow cytometry with Sysmex XN-20 (Sysmex Corporation, Japan). For this purpose, hematimetry samples from 12 patients, the viral load of which had been previously analyzed using specific EDTA plasma sample with the Cobas 6800 analyzer (Roche Diagnostics, Germany) were selected: three blood samples (c1 to c3) had a viral load higher than 6 logs, and in nine blood samples (i1 to i9), the viral load was undetectable. After selection of samples according to viral load, they were reprocessed in the Sysmex XN-20 analyzer in the same analytical series, following this sequence: c1-c2-c3/i1-i2-i3/c1-c2-c3/i4-i5-i6/c1-c2-c3/i7-i8-i9, which maximized the risk of carryover from cases with a high viral load to those with an undetectable viral load: three samples with a high viral load followed by three samples with an undetectable viral load. Afterwards samples were centrifuged, and HCV RNA was determined in those samples containing a previously undetectable viral load.

To assess the stability of the HCV RNA viral load, 29 samples with a viral load higher than 4 logs were selected. The viral load was determined the same day it was taken, at 24 and 72 h after being stored at 4 to 8°C. Results obtained were compared using the Wilcoxon test.

### Liver fibrosis assessment.

The degree of liver fibrosis was assessed using the FIB-4 index (12) in serum samples of HCV RNA-positive patients during March to December 2018. The FIB-4 index is currently considered a valid tool for assessing the degree of fibrosis (10, 13), and allows identification of clinically significant fibrosis (≥F3) at the threshold of an FIB-4 score of >3.25 (4). The relationship between patient’s age, degree of fibrosis, and HCV RNA was also investigated. Means of the FIB-4 index obtained for the different groups were compared using the Student's *t* test. For the two factors being investigated (age, detectable viral load, and both), the odds ratio (OR) for the presence of advanced fibrosis was estimated.

## RESULTS

### Sample suitability and integrity for reflex testing.

Of the nine samples without a tested detectable viral load, seven remained free of HCV RNA, while amounts lower than the quantification limit of the analytical process were detected in two samples (<15 IU/ml). Regarding the integrity of HCV RNA over time, the Wilcoxon test did not reveal any significant differences in its levels throughout the period of conservation of the samples.

### Result of the six-step protocol with active recommendation.

Between January 2015 and March 2018, anti-HCV antibody determination was performed in 140,329 patients from primary care centers, of which 5,436 (3.9%) tested positive for anti-HCV. In 2,716 (50%) anti-HCV-positive patients, the laboratory included a recommendation to perform an HCV RNA analysis in the printed and online result reports, which was followed in 1,412 cases (52% of all recommendations), and the determination of HCV RNA was therefore requested and performed. Of all cases in which the recommendation was omitted, 986 (36%) corresponded to HCV patients with known infection, whereas in 327 (12%) cases, the reason for omitting HCV RNA determination was unknown.

### Result of implementing the one-step protocol.

During the entire period analyzed (2017 to 2018), a total of 128,721 cases were collected (Table 1). During the six-step protocol period, omitted HCV RNA analyses ranged from 8% to 82% of all anti-HCV-positive patients. The setting in which less HCV RNA tests were requested for anti-HCV-positive patients in this period was drug treatment centers (CAS) (82%), followed by primary care settings(28%). After implementation of the one-step protocol (based on reflex testing), the percentage of HCV RNA tests that were omitted in anti-HCV-positive patients was ≤11% in all settings. In the case of drug treatment centers and primary care settings, the absolute percentages of omitted HCV RNA analyses decreased 76.4% (from 81.5% to 5.1%) and 20.2% (from 28.4% to 8.2%), respectively. Table 1 shows the number of cases with detectable HCV RNA, and Table 2 shows the range and median of HCV RNA levels for each group.

**TABLE 1:** Total cases analyzed under the traditional protocol (from January 2017 to February 2018) and the reflex testing (one-step) protocol (from March 2018 to December 2018)

Setting^*a*^	No. (%) with result by:									
	6-step protocol					Reflex testing protocol				
	*n*	aHCV positive	HCV RNA analysis^*b*^	HCV RNA analysis omitted^*c*^	Detectable HCV RNA	*n*	aHCV positive	HCV RNA analysis^*b*^	HCV RNA omitted^*c*^	Detectable HCV RNA
Hospital care	20,446	2,583 (12.6)	2,998	1,697 (8.3)	979 (32.7)	10,740	616 (5.7)	831	70 (11.4)	191 (23.0)

Non-hospital care	62,340	2,612 (4.2)	1,885	776 (29.7)	752 (39.9)	35,195	1,126 (3.2)	1,093	82 (7.3)	458 (41.9)
CAS	1,335	211 (15.8)	39	172 (81.5)	21 (53.8)	1,052	235 (22.3)	245	12 (5.1)	132 (53.9)
Itinerant	2,644	383 (14.5)	360	35 (9.1)	199 (55.3)	401	53 (13.2)	57	2 (3.8)	30 (52.6)
Primary care	57,830	2,010 (3.5)	1,480	570 (28.4)	530 (35.8)	33,399	831 (2.5)	784	68 (8.2)	295 (37.6)
Other	531	8 (1.5)	6	2 (25.0)	2 (33.3)	343	7 (2.0)	7	0	1 (14.3)

Total	82,786	5,195 (6.3)	4,883	2,476 (47.7)	1,731 (35.4)	45,935	1,742 (3.8)	1,924	152 (8.7)	649 (33.7)

a “Primary care” represents the general population seeking treatment in a community health center, “CAS” represents drug treatment centers, “Hospital care” represents hospitalized patients, hospital consultations, and an external site specialized in imported and sexually transmitted diseases, “Itinerant” represents patients who for different reasons are sequentially or even simultaneously treated by different units and thus are shared by the different groups mentioned above, and “Other” represents prisons and children’s care homes.

b The number of HCV RNA analyses may be greater than the number of aHCV (anti-hepatitis C virus antibody)-positive analyses because some patients were previously known to be aHCV positive and serology was not repeated.

c The percentage of omitted HCV RNA analyses was based on the number of patients with aHCV without HCV RNA determination (either before or after) at the time of data collection. In the case of the reflex testing protocol, all omitted analyses were due to technical issues.

**TABLE 2:** HCV RNA levels in cases detected by the reflex testing (one-step) protocol (from March 2018 to December 2018)

Setting	No. of patients with detectable HCV RNA	HCV RNA level (IU/ml) in group		
		Minimum	Maximum	Median
Hospital care	191	<15	7.4 × 10^7^	2.2 × 10^6^

Non-hospital care	458			
CAS^*a*^	132	16	2.1 × 10^7^	1.2 × 10^6^
Itinerant	30	47	1.2 × 10^7^	1.5 × 10^6^
Primary care^*b*^	295	114	4.6 × 10^7^	1.7 × 10^6^
Other	1	NA^*c*^	NA	NA

Total	649			

a Four cases with an HCV RNA level of <15 IU/ml were observed.

b One case with an HCV RNA level of <15 IU/ml was observed.

c NA, not applicable.

Of the 1,924 anti-HCV-positive patients with HCV RNA determination in the second analysis period (i.e., after implementing the reflex testing protocol), 1,034 (53.7%) had been requested by the physician, whereas 890 (46.3%) were performed as reflex testing despite not having been requested (Table 3). The result of the HCV RNA analysis was provided in a mean of 2 days after anti-HCV determination. In the primary care setting, of the 295 patients with detectable HCV RNA during this period, the reflex testing had been performed on 163 (55.3%), despite the fact that the doctor had not requested it. In the case of drug treatment centers, positive HCV RNA identified thanks to reflex testing amounted to 60.6% of the total.

**TABLE 3:** HCV RNA detected during the period from March to December 2018 based on the source of the analysis request

Setting	No. (%) of results with:			
	HCV RNA analysis requested		HCV RNA analysis not requested (reflex testing only)	
	Determination of HCV RNA (*n* = 1,034)^*a*^	HCV RNA detected	Determination of HCV RNA (*n* = 890)^*a*^	HCV RNA detected
Hospital care	704 (84.7)	161 (22.9)	127 (15.3)	30 (23.6)
				
Non-hospital care	330 (30.2)		763	
CAS	97 (39.6)	52 (53.6)	148 (60.4)	80 (54.1)
Itinerant	23 (40.4)	16 (69.6)	34 (59.6)	14 (41.2)
Primary Care	206 (26.3)	132 (64.1)	578 (73.7)	163 (28.2)
Other	4 (57.1)	1 (25.0)	3 (42.9)	0

a Percentages are over the total number of assessments in a given setting.

### Liver fibrosis evaluation.

All data necessary to establish the FIB-4 index were available in our LIS in 869 (77%) of 1,126 anti-HCV-positive outpatients analyzed between March and December 2018. Both age and the presence of HCV RNA were significantly associated with the FIB-4 score and the three degrees of fibrosis: advanced, absence, and undetermined (Table 4). The ORs of advanced fibrosis associated with age of ≥65 years and detectable levels of HCV RNA were 5.2 (95% confidence interval [CI], 3.3 to 7.9) and 3.6 (95% CI, 2.2 to 5.8), respectively. Taken together, patients aged 65 years or older with detectable levels of HCV RNA had an OR of 5.92 (95% CI, 3.4 to 10.4) of having advanced fibrosis.

**TABLE 4:** Degree of fibrosis of anti-HCV-positive patients analyzed during the period from March to December 2018 according to age and positive/negative HCV RNA

Parameter^*a*^	Result for parameter by:					
	Age		*P*	HCV RNA		*P*
	≥65 yr (*n* = 183)	<65 yr (*n* = 686)		Negative (*n* = 399)	Positive (*n* = 402)	
FIB-4 score, mean (95% CI)	3.09 (2.73–3.44)	1.57 (1.45–1.69)	<0.001	1.52 (1.39–1.66)	2.27 (2.05–2.49)	<0.001

Fibrosis grade, *n* (%)						
Advanced fibrosis	52 (28.4)	49 (7.1)		23 (5.8)	71 (17.7)	
Absence of fibrosis	24 (13.1)	447 (65.2)	<0.0001	259 (64.9)	176 (43.7)	
Undetermined	108 (58.8)	190 (27.7)		117 (29.3)	155 (38.6)	

a CI, confidence interval. The degree of fibrosis was defined based on the FIB-4 score according to the following cutoffs: >3.25 for advanced fibrosis (65% positive predictive value and 97% specificity) and <1.45 for absence of fibrosis (90% negative predictive value and 70% sensitivity). Scores between 1.45 and 3.25 were considered undetermined.

## DISCUSSION

The results from this study show how the application of a viral load reflex testing (or one-step) protocol for the diagnosis of HCV infections in a central laboratory allows an outstanding increase in diagnostic rates, particularly in the primary care setting, which in our discipline entails the main entry point for new cases of HCV infection, and drug treatment centers, where hepatitis C has the highest prevalence in our area (21%).

To implement this screening protocol, it was essential to first ensure the suitability of the sample in which the viral load was to be determined. The fact that the flow cytometry analyzer keeps samples capped while processing them—thus preventing contact with aerosols and environmental RNAses—strongly reduced the likelihood of contamination and deterioration of viral RNA. Furthermore, the probability of a sample free of HCV RNA matching a sample with a detectable viral load in the same analytical sequence was in fact much lower than the probability of both samples coexisting in the same anti-HCV antibody determination sequence in the serology analyzer (even taking into account the low probability in the Cobas 8000 analyzer due to the use of disposable tips). Despite the *a priori* favorable conditions to implement the reflex testing protocol, an occasional error in the washing system of the blood count analyzer could cause minimal contamination, like that observed in two of the nine analyzed samples in which HCV RNA was detected, although in amounts below the quantification limit of 15 IU/ml. In this regard, it is important to mention that, in naive patients, viral loads have been previously reported to be >3 logs in a very high proportion (EASL guidelines) or even >2 logs based on our own experience (14). In fact, in the cases analyzed in this study, after application of the HCV RNA reflex testing, only nine cases (one in the primary care setting and eight in the CAS setting) showed HCV RNA levels of <2 logs. Among them, only five cases with levels of <15 IU/ml (four in CAS and one in primary care) were observed, which, after further investigation, were all related to antiviral treatment. Hence, a value of <15 IU/ml should be considered irrelevant in the context of a new diagnosis (10). Still, faced with this potential risk, the need to evaluate the viral load in a new sample in cases with an HCV RNA level of ≤2 logs was included in the protocol (Fig. 2). Given the sampling path in our area, the sample collected for blood count analysis seems to be optimal for viral load reflex testing; however, other type of samples processed before could be used, provided that their adequacy (i.e., stability and lack of contamination) has been assessed. This possibility is also indicated in our recommended algorithm, which in fact is currently being implemented in all Catalonia.

In our experience, the traditional protocol, in which patients needed six visits for a full diagnosis (Fig. 1A), had a low ratio of HCV RNA confirmatory tests, especially in the ambulatory setting (non-hospital care), where the HCV RNA analysis was omitted in 9 to 82% of cases, depending on the source of the sample. This omission was particularly worrisome in the primary care setting, where HCV RNA analysis was omitted in the 28% of the around 2,000 anti-HCV-positive subjects from a total population of 58,000 individuals. These data confirm the low percentage of HCV RNA confirmatory test performance (46% to 73%) observed in protocols with multiple visits (8). Improvements observed after implementing the new one-step protocol were particularly notorious in the ambulatory setting (outpatients), with increases from 18% to 95% of HCV infection cases in CAS and from 72% to 92% in primary care centers: the contributions of reflex testing to the identification of new cases in these care settings were 54% and 28%, respectively. The spectacular increase in diagnostic rates in drug treatment centers is particularly relevant in terms of public health, due to the high prevalence of HCV infection among drug users. An effective diagnosis in this setting enables starting antiviral treatment, likely to result in HCV curation (15), a key cornerstone for HCV infection elimination (2, 7). However, a low rate of anti-HCV-positive cases remain undiagnosed due to lack of viral load testing; in our case, most are due to lack of hematimetry requests, meaning no samples for HCV RNA testing. In these cases, the validation of other types of samples, such as one’s own sample previously processed for serology, could be used for viral load reflex testing. However, this would require conducting a prior study ensuring its use, such as that performed here regarding hematimetry tubes.

Aside from the benefits of this technique at the level of public health, reflex-testing-based diagnosis means fewer visits to the doctor and unnecessary blood draws. This, in turn, translates into not only more comfortable patients, but also fewer subjects lost to follow-up and a shorter delay time for diagnosis and referral to a specialized center to receive treatment. It is noteworthy that the reflex testing protocol provided a diagnosis within a mean of 2 days, in contrast with the six-step protocol, which lasted weeks—or even months—due to administrative barriers to scheduling extra appointments. In addition to a shorter time for diagnosis, previous studies have found that the application of similar protocols are cost-effective (16). In this regard, it is important to highlight that the cost of a viral RNA determination (less than 30 euros in our laboratory) is much lower than the cost of a physician appointment and a blood extraction, so overall cost reduction may be of note. Furthermore, our algorithm includes request containment tools, such as avoiding duplicated determinations for patients with previous positive anti-HCV test results.

Alternative methods for testing for HCV infection outside of a central laboratory, such as the rapid diagnostic test (RDT) with capillary finger blood (e.g., OraQuick HCV) (17), have higher costs (i.e., 15 euros per test or higher) and poorer outcomes (<98% of correspondence). However, these methods can be beneficial in areas or population groups where the implementation of central laboratory strategies is unreliable (e.g., active drug users). Notwithstanding the benefits of other methods, central laboratory methodologies are strongly recommended by international guidelines for populations outside of the normal health system as well, thus allowing the implementation of one-step strategies, irrespective of the type of sample used. An example of an alternative sampling has been described by Lazarus et al., who implemented a one-step diagnosis strategy using whole blood sampled on dried blood spots (18). In this regard, the use of the GenXpert platform (Cepheid, Sunnyvale, CA, USA), which has shown an extremely optimal correlation with high-throughput systems (19, 20), represents an adequate alternative for HCV viral load determination when it is not possible to perform it in a central laboratory. This is the case of special populations like, for instance, active intravenous drug users, in which it seems advisable to conduct any viral load testing at the same center and start antiviral treatment as soon as possible. In these cases, the extreme high prevalence of HCV infections (>70% seroprevalence according to a recent meta-analysis) (21) would even make direct screening of viral loads cost-effective by means of GenXpert technology (Cepheid) in the same drug treatment center. However, in addition to the possibility of a concomitant determination of blood count and HCV RNA, HCV diagnosis with venipuncture for standard blood sampling in central laboratories had the advantage of the possibility of assessing patients’ liver fibrosis, which is necessary for their management in case of liver cirrhosis or advanced fibrosis (10, 22). In this regard, an HCV diagnosis strategy encompassing the FIB-4 index assessment promotes the patient’s start of treatment, while raising awareness on patients with advanced fibrosis. Clinical Practice Guidelines recommend combining noninvasive biomarker-based measurements, such as the FIB-4 index, with physical examinations, such as transient elastography (TE); however, not all sites have this technology (23). Aside from the high positive and negative predictive values of FIB-4 for the diagnosis of advanced fibrosis and cirrhosis (24, 25), combining the FIB-4 index and the TE examination in sequence increases the accuracy of the diagnosis of the fibrosis stage (26). The results found in this study show the high probability of advanced fibrosis in patients with a detectable viral load (17%) and those over 65 years of age (28%). These data provide a global vision of the significance of the degree of liver fibrosis in these patients and the importance of diagnosing the state of the infection early.

The extremely positive development of hepatitis C therapies is being darkened by the sad reality that the majority (80%) of people with chronic HCV infections remain undiagnosed (2). Achieving the WHO objective of eliminating viral hepatitis as a major public health threat by 2030 will require urgent strategies facilitating a massive scale-up of timely diagnoses as an essential step to drive infected individuals to treatment. It must be kept in mind that late diagnosis and treatment of chronic HCV infections may result in severe consequences for the health of both the individual and the population. In this regard, 30% of HCV-infected patients with late diagnosis will benefit less from achieving viral elimination, as well as those with advanced liver disease who are at high risk of developing decompensated cirrhosis, portal hypertension, and hepatocellular carcinoma (2, 27, 28). The occurrence of late chronic hepatitis C should be assumed as a failure of the health system to timely diagnose infected individuals. Overcoming this issue will require to promote early screening using a strategy as effective and simple as possible. Since the reflex testing strategy is driven by—and depends on—a central laboratory, patient treatment start (including the assessment of liver fibrosis) can be achieved without implementing sophisticated and expensive screening programs.

Our analysis has the typical limitations of an observational study in which all data have been obtained from a record designed with purposes of care and laboratory operations management. This design has allowed us to bring together data from 128,721 patients, providing an accurate perspective of the population reality; however, no patient social-demographic and clinical information could be collected, which could have improved the study. Furthermore, we must bear in mind that the scope of the results may be conditioned by the organization of the health care system and the laboratory itself, so, depending on the context, the contribution of the one-step protocol to the diagnosis of new cases of HCV could be lower or even higher.

In summary, our study shows the potential that implementing a diagnostic protocol based on reflex testing in a central laboratory may have to identify new cases of HCV infection significantly faster (2 days instead of weeks or months) and with much higher effectiveness than the traditional six-step process. Although results must be interpreted within the context of laboratory practice and the health care system of each area, our experience encourages the implementation of this type of analyses, which may notably contribute to the elimination of HCV infections. In this regard, it seems evident that universal screening programs for HCV infection represent the optimal solution to detect hidden HCV cases and start treatment, thus meeting the WHO aims. Given that the general population is mostly managed in the primary care setting, one-step diagnosis programs can effectively cover a high proportion of the general population at a low cost. Overall, the cost-effectiveness of implementing hepatitis C screening programs in the general adult population has been demonstrated (29). The availability of high-performance analytical platforms like Cobas 8000 and Cobas 6800, used in our laboratory and able to process thousands of samples in a normal working day, strongly facilitates the implementation of such screening programs. However, additional strategies for groups at high risk, very frequently not linked to the health system, might be necessary to reach the entire population of HCV-infected individuals. In this regard, the accurate and simple point-of-care testing platforms, specially the HCV finger-stick assay in the GenXpert analyzer (Cepheid), represent a very interesting alternative for these special populations.
